# Tracking the Position of the Heart From Body Surface Potential Maps and Electrograms

**DOI:** 10.3389/fphys.2018.01727

**Published:** 2018-12-03

**Authors:** Jaume Coll-Font, Dana H. Brooks

**Affiliations:** ^1^Computational Radiology Laboratory, Children's Hospital, Boston, MA, United States; ^2^Harvard Medical School, Boston, MA, United States; ^3^Signal Processing, Imaging, Reasoning, and Learning (SPIRAL) Group, Electrical and Computer Engineering Department, Northeastern University, Boston, MA, United States

**Keywords:** electrocardiographic imaging, inverse problems, respiration, ECGI, forward problem, electrocardiography, heart tracking

## Abstract

The accurate generation of forward models is an important element in general research in electrocardiography, and in particular for the techniques for ElectroCardioGraphic Imaging (ECGI). Recent research efforts have been devoted to the reliable and fast generation of forward models. However, these model can suffer from several sources of inaccuracy, which in turn can lead to considerable error in both the forward simulation of body surface potentials and even more so for ECGI solutions. In particular, the accurate localization of the heart within the torso is sensitive to movements due to respiration and changes in position of the subject, a problem that cannot be resolved with better imaging and segmentation alone. Here, we propose an algorithm to localize the position of the heart using electrocardiographic recordings on both the heart and torso surface over a sequence of cardiac cycles. We leverage the dependency of electrocardiographic forward models on the underlying geometry to parameterize the forward model with respect to the position (translation) and orientation of the heart, and then estimate these parameters from heart and body surface potentials in a numerical inverse problem. We show that this approach is capable of localizing the position of the heart in synthetic experiments and that it reduces the modeling error in the forward models and resulting inverse solutions in canine experiments. Our results show a consistent decrease in error of both simulated body surface potentials and inverse reconstructed heart surface potentials after re-localizing the heart based on our estimated geometric correction. These results suggest that this method is capable of improving electrocardiographic models used in research settings and suggest the basis for the extension of the model presented here to its application in a purely inverse setting, where the heart potentials are unknown.

## 1. Introduction

Subject-specific solutions to the forward problem of electrocardiography, that is, producing a mathematical model that can estimate body surface potential maps (BSPMs) from knowledge of cardiac electrical activity and an individualized thoracic volume conductor model, is important in a number of settings. These include tools for understanding and pedagogy about the ECG (van Oosterom and Oostendorp, 2004), methods to guide interventions such as ablation through simulation (Trayanova, 2011), and solutions to the corresponding inverse problem of characterizing cardiac electrical activity from body surface measurements (commonly known as ElectroCardioGraphic Imaging, ECGI) (Pullan et al., 2010). The forward solution is known, from electrostatic theory, to be quasistatic and to depend only on the geometry of the torso and of the organs inside of it and their respective conductivities. Moreover, under specific assumptions about the electrical source models on the heart this relationship can be accurately modeled as linear. However, forward solutions depend on the underlying geometry, since it specifies the boundary conditions for the underlying partial differential equation, and even though this sensitivity is well-behaved, it is particularly critical when solving the ECGI inverse problem, since that inverse problem is ill-posed and very sensitive to errors in the forward model. Thus methods to improve forward modeling have received considerable attention in the ECGI community (Coll-Font et al., 2016a) and there is open discussion about, for example, what is the “best” forward model to use, which source models better characterize the electrical activity of the heart, how many, and which, organs should be included in the geometry, and how their respective conductivities should be estimated (Ferguson and Stroink, 1997; Ramanathan and Rudy, 2001a,b; Weber et al., 2011; Jones et al., 2013; Bear et al., 2015; Dehaghani, 2015; Potyagaylo et al., 2016; Punshchykova et al., 2016).

However, there is an additional challenge that is often ignored in this discussion: the positions of the organs within the torso, including the heart, are not static; rather they vary due to respiration and to changes in position of the subject. The sensitivity of forward solutions to these variations have been studied (Geneser et al., 2008; Swenson et al., 2011) but overcoming it remains challenging. It cannot be resolved a priori with better segmentation and it is not always possible to address by procedural mechanisms such as requiring the patient to retain breath-hold position. This challenge also appears in many phantom and animal experiments, such as validation of ECGI (MacLeod et al., 2000; Erem et al., 2014; Bear et al., 2015; Cluitmans and Volders, 2017), where the true position of the heart is not only unknown but subject to several experimental uncertainties and might change from beat to beat.

Here we address this limitation by attempting to use the changes in ECG due to changes in heart position—in other words, the manifestation of the problem itself—as the source of a solution. Specifically, since changes in position of the heart produce changes in the distribution of body surface potentials, we investigate whether that very variation can be used to track these positional changes and thus “correct” the forward model. In this paper we describe a method to estimate and correct for the translation and rotation of the heart for each heartbeat. We evaluate our accuracy in doing so by examining geometric accuracy in a controlled simulation and to what extent estimating these geometry changes leads to decreased errors in accuracy of both forward model body surface potential calculations and of associated inverse solutions. We report results for both synthetic experiments and in the context of three different physical experiments carried out with canine hearts suspended in a human torso-shaped tank phantom.

Our work builds on previous reports relating changes in geometry—and thus changes in forward models—to changes in the ECG. The classical studies described the changes in ECGs from patients as a function of the respiratory cycle. These studies showed that the changes can be characterized as a continuous displacement of the maxima and minima of the body surface potential maps (BSPM) (Amoore et al., 1988), have different effects along the PQRST sequence (Adams and Drew, 1997; Madias, 2006), and are subject specific (Nelwan et al., 2001). More systematic experiments on animal models and synthetic data provided methods to estimate the average BSPM and the variance that can occur due to movement of the heart in a subject (MacLeod et al., 2000; Swenson et al., 2011). More recently, cardiac magnetic resonance imaging allowed characterization of the relationship between standard clinical metrics of the ECG and changes in the heart geometry (Lyon et al., 2017) and, specific to ECGI, Cluitmans et al. explored the effects of these geometry errors on inverse solutions (Cluitmans and Volders, 2017).

Closer to our work, there have been a few reports attempting to track the changes in position of the heart using BSPM. Shvelikhova et al. estimated the vertical position of the heart by characterizing its electrical activity with a moving dipole whose position was tracked from the ECG (Svehlikova et al., 2011). Recently, Rodrigo et al. proposed to pre-compute a set of candidate forward models and then used a metric derived from the L-curve in Tikhonov regularization to select the “best” candidate forward model (Rodrigo et al., 2017, 2018). These approaches produce an optimization problem to be solved that is computationally tractable, but require pre-computation of a set of forward models from which to choose, or have very strong assumptions about the form of the source and geometry models, which may limit their generalization. In any case they are complementary to the method described here.

In this paper we describe the formulation and experimental validation of our approach. Specifically we reverse the role of geometric assumptions and cardiac surface potentials with respect to the traditional inverse problem of electrocardiography; instead of estimating the electrical sources of the heart from the ECG measurements and the geometry, we correct the geometric model (e.g., translations and rotations of the heart) assuming knowledge of the electrical measurements on both the heart and the body surface (Coll-Font, 2016; Coll-Font et al., 2016b, 2017). Direct application of this approach is relevant to a variety of phantom and animal studies where measurements can be made on both surfaces. A future extension might allow use of only a limited set of heart surface potentials such as those acquired during catheter procedures. Future application to ECGI would require estimating both heart surface potentials as well as geometry correction parameters and, while preliminary results are positive, success clearly depends on establishing the validity and limitations of the geometry correction approach in its own right, which we attempt to do in the current paper.

In the following we describe in section 2 how our method is applied to this type of data, we present the experiments we used for validation and the corresponding results obtained sections 3 and 4, respectively, discuss their implications in section 5, and summarize our conclusions in section 6.

## 2. Methods

The work presented here assumes the availability of a nominal discretized surface model for the heart and torso geometries; however we do not assume that the position of the heart within the torso is accurately known. The torso is treated as homogeneous in the experiments reported here but this is not required by the method as long as the geometry of any other organs included in the model is known. The electrical activity of the heart is modeled as a time series of potentials on a surface that surrounds the ventricles. (Again, extension to alternative source models would be straightforward). We further assume that we have available potentials measured on the heart and torso surfaces at multiple time instances, denoted *x*_*b*_(*t*) and *y*_*b*_(*t*), respectively, where we index time within a beat by *t* and heartbeats by *b*. Under these assumptions, the electrical forward model is represented in the form of a forward matrix (denoted *A*) with the heart at some reasonable position in the torso volume. We refer to the matrix *A* that corresponds to this nominal position in the sequel as the “nominal” forward solution. We note that this nominal position will be used as a starting point in our iterative algorithm but that there is no requirement that it be particularly accurate; we assume that a nominal model computed from imaging scans will provide a reasonable “initial guess.” With these assumptions, we have the following putative nominal relationship between heart and body surface potentials (MacLeod and Buist, 2010):

(1) $$\begin{array}{r} y_{b}\left(t\right)=Ax_{b}\left(t\right). \end{array}$$

Our work assumes that the heart can change position at every heartbeat *b*; thus Equation (1) must be extended to reflect the corresponding changes in the forward model. We postulate an equivalent sequence of forward models, *A*(*p*_*b*_), parameterized by a joint position and orientation parameter vector *p*_*b*_, that relates the position of the heart to the measured body and heart surface potentials.

(2) $$\begin{array}{r} y_{b}\left(t\right)=A\left(p_{b}\right)x_{b}\left(t\right). \end{array}$$

**Figure 1:**
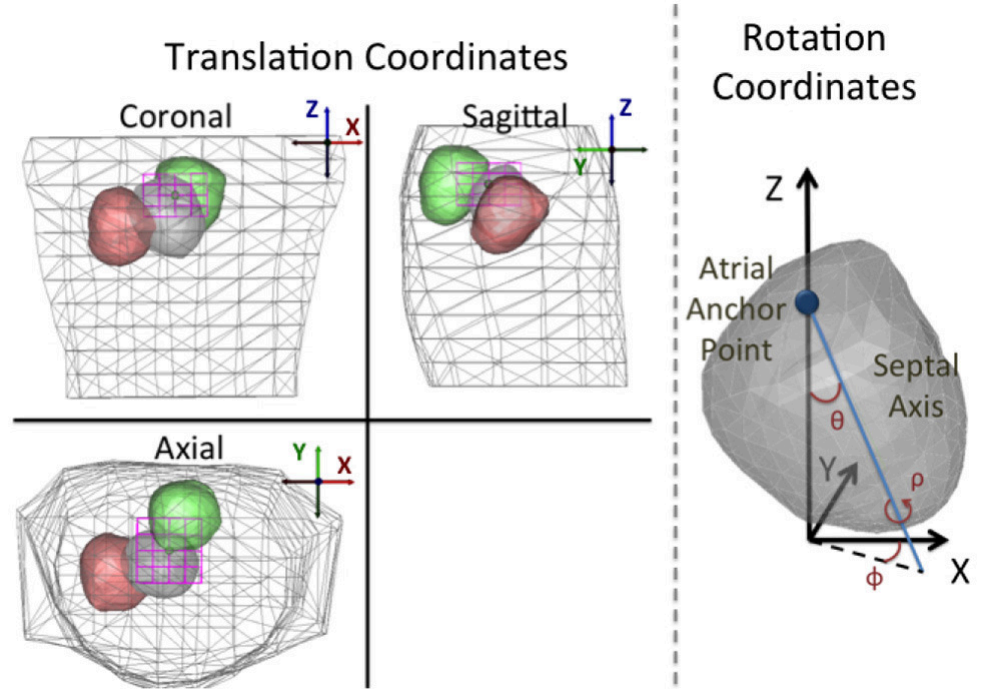
Depiction of the parameterization of the translation and rotation coordinates. **Left**: Standard orthogonal projection views of the torso and heart for three different position/angle combinations. Translation coordinate axes are defined following the convention presented in the EDGAR repository (Aras et al., 2015). The magenta box shows the translation bounding box for the assumed anchor point. As described in the text, the translation and angle constraints are defined such that the heart geometry can never intersect the torso surface. **Right**: Rotation angles defined on the heart. Pitch (θ): the angle formed between the Z axis and the septal axis. Yaw (ϕ): the angle that the septal axis projected on the X/Y plane forms with the X coordinate. Roll (ρ): the rotation of the heart around the septal axis.

We chose a specific parameterization that effectively characterizes the expected translation and rotation of the heart due to respiration. In particular, although the respiratory movement of the heart is subject specific, there are common features that can be leveraged to describe it: the heart translates vertically and undergoes rotation around a tethering point on the left atrium (Netter, 2006; Coll-Font et al., 2011; Aras et al., 2015). Based on this description, we defined translation parameters using a standard coordinate system (from the EDGAR database formulation; Aras et al., 2015), and defined rotations with respect to two anatomical references: one is an anchor point placed at the centroid of the atria and the other a septal axis that crosses the heart through the septum from that atrial anchor point to the apex (see Figure 1 for illustration). Based on these references the rotation angles are defined as:

- Pitch (*θ*): the angle formed between the Z axis and the septal axis.
- Yaw (*ϕ*): the angle formed between the septal axis projected on the axial plane and the X axis.
- Roll (*ρ*): the rotation of the heart around the septal axis.

Once this parameterization is defined, the generation of forward matrices requires moving the heart to the position and rotation described by the parameters *p*_*b*_ and then computing the corresponding forward matrix with an appropriate forward solver.

The implicit function *A*(*p*_*b*_) defined in this formulation is a manifold in the space of matrices. It is a non-linear, continuous and smooth function—i.e., small variations in the position of the heart will lead to small changes in the forward matrix—and hence it can be used in an optimization framework. Specifically, we need to solve an optimization problem that searches for the translation and rotation parameters of the heart—within some reasonable bounds—that minimize the error between ECG potentials synthesized using Equation (2) and the potential measurements on both surfaces. The main assumption in this optimization problem is that the dominant error observed in the synthesized potentials is caused by errors in the position and orientation of the heart and that other sources of error can be modeled as additive white Gaussian noise or are negligible. This results in a non-linear least-squares problem over the six-dimensional vector *p*_*b*_, restricted in each dimension to a hyper-rectangle as in Equation (3).

(3) $$\begin{array}{r} \begin{array}{cc} \underset{p_{b}}{\min} & \underset{t}{\sum }||y_{b}\left(t\right)-A\left(p_{b}\right)x_{b}\left(t\right)|{|}_{2}^{2}\\ \;st.p_{b}\in \Xi \end{array} \end{array}$$

were the norm is taken over both time and space. The hyper-rectangle constraint, illustrated in Figure 1 and denoted as Ξ, was defined a priori to prevent any intersection between the heart geometry and the torso surface.

This constrained non-linear optimization problem can be solved with any off-the-shelf solver. In the particular case of these experiments, we used MATLAB's default iterative solver of the fmincon function^1^, which implements an Interior-Point method with numerically approximated gradients and Hessians.

We note that in this problem the solution is constrained to lie on a non-linear manifold induced by the parameterization in a high-dimensional subset of matrices restricted to be approximations to the underlying PDE. Thus the problem is difficult to analyze mathematically and, in particular, we have no guarantee that it is well-posed^2^. However these restrictions imposed on the solution are highly constraining. Thus we believe it is reasonable to hypothesize that solutions are stable. Our experimental results, as reported below, support this hypothesis.

## 3. Experiments

The immediate purpose of the work presented here is to determine if in fact the translation and rotation of the heart estimated by our proposed algorithm can reduce the effects of model errors present in the numerical and physical experiments we studied. To that purpose, we both created synthetic data to model respiratory movement and employed data recorded during three different torso-tank canine experiments conducted at the Cardiovascular Research and Training Institute (CVRTI), University of Utah.

**Synthetic Data:**

We generated synthetic respiratory motion influenced data using the **sock2** heart and body surface geometry described below and the potentials from a single beat on the epicardial surface recorded with an electrode mesh, also as described below. Starting from a nominal position inside the homogeneous torso model, we moved the heart to 10 different positions and orientations following a respiratory-like trajectory described in (Coll-Font et al., 2011).^3^ For each of these positions/orientations, we synthesized one heartbeat of BSPM using a forward model computed from that geometry using the Boundary Element Method (BEM) provided with the SCIRun software system (SCI-Institute, 2014)^4^ and added independent Gaussian noise to achieve an SNR = 30 dB. We then fed the epicardial and body surface data and the (incorrect) nominal forward model into our algorithm and attempted to estimate the corrected position and orientation of the heart for each of the 10 beats. We repeated this procedure 10 times for different realizations of the pseudorandom noise.

**Experimental Data:**

Data was generously provided to us from canine experiments that had been carried out for previous studies with applicable IACUC approval. These experiments consisted of unipolar recordings of potentials on or near the epicardial surface of an explanted canine heart measured simultaneously with similar recordings on the surface of a torso-shaped tank in which the heart was suspended (MacLeod et al., 1995a,b). The tank was filled with conductive medium. The homogeneous conducting medium and the availability of unipolar recordings on both surfaces match the assumptions described in section 2. During the experiment, the suspended heart was kept alive through retrograde perfusion with blood from a “support” animal that provided circulation through the left anterior descending (LAD) artery. This setting allowed the experimenters to both pace the heart at different locations, through electrodes placed intramurally or on the heart surface, and to induce ischemia by either accelerating the pacing rate or by occluding the LAD. At the end of the experiment, the heart was vertically raised from its position during the experiment and the 3D coordinates of several electrodes were digitized and used to register the heart geometry to its estimated position within the tank. This registration procedure includes the measured vertical displacement of the heart, thus assuming that it was raised and lowered into the tank with no inclination with respect to the tank geometry. Note that buoyancy effects and tension from electrical cables and blood supply tubing might introduce error in the geometry that is not corrected by the registration.

We used this measured geometry to construct our nominal forward model, again computed with the SCIRun BEM solver, and then used the recorded potentials on both surfaces over multiple beats in the method described above to estimate the position and orientation of the heart on a beat-by-beat basis.

**Figure 2:**
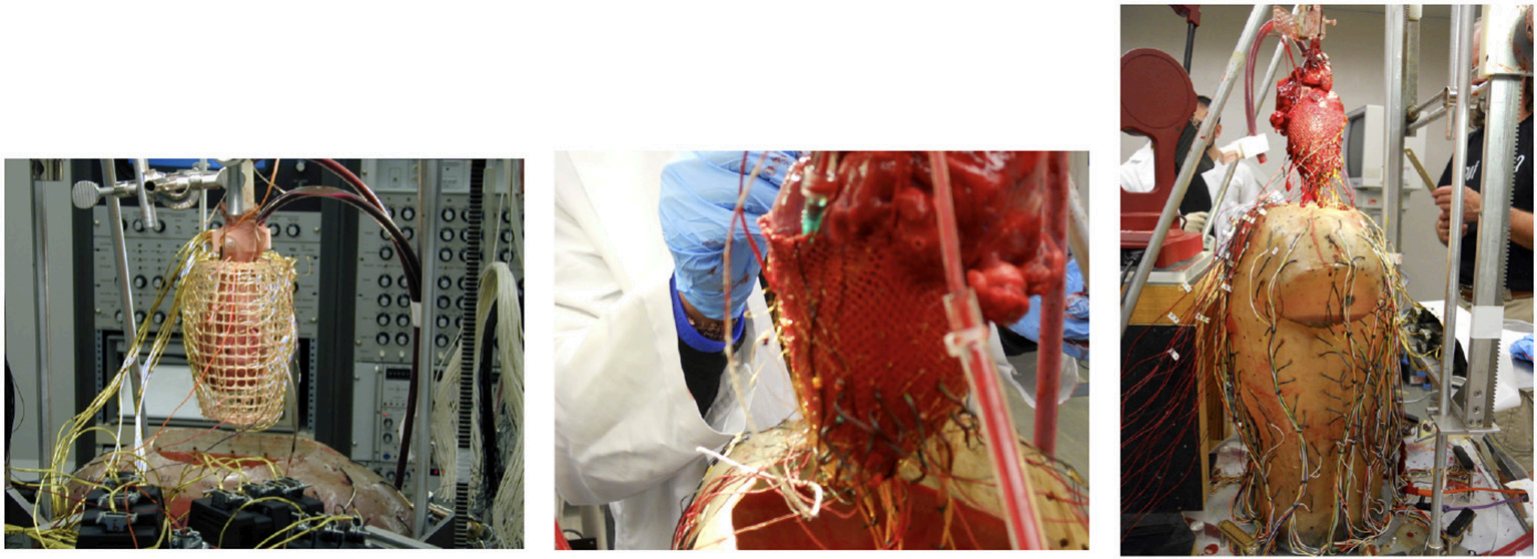
Pictures of the recording devices on the heart using a cage geometry **(Left)**, a sock geometry **(Middle)**, and the entire experimental apparatus **(Right)**.

Two different experimental methods were used to record the heart surface potentials. In one experiment, the heart was enclosed in a small wire cage with electrodes on the cage itself. In the other two, a mesh, or “sock,” that had been wired with a large number of electrodes was stretched around the heart surface and tightly tied around the ventricles. Figure 2 shows visual examples of the apparatus during an experiment. These two heart surface potential measurement approaches have respective benefits and drawbacks. The cage electrodes are placed at some distance from the heart and thus measure its electrical activity over a rather broad area, resulting in considerable spatial smoothing of potential distributions compared to measuring them directly on the heart surface. In contrast, the sock electrodes measure more local, spatially resolved, electrical activity. However, the sock is flexible and hard to fix in place on the heart surface, and its geometry is sensitive to contraction, swelling of the heart, and any other changes in heart shape, and in addition may be displaced during any interventions. Moreover the sock moves as the heart moves, and the heart was not securely fixed in a consistent, repeatable, and accurately measurable location during the entire experiment. In contrast the position of the cage is easier to both measure and maintain throughout the experiment. Thus sock recordings are more prone to error in the geometric model, including time-varying errors, than are cage recordings. Another technical limitation of the sock is that the electrodes only cover the ventricles, leaving an opening around the atria. In order to use BEM forward solvers, the heart geometry must form a closed surface, thus requiring the generation of “extra” nodes closing the geometry for which there are no actual measurements available. By contrast the cage has electrodes that completely surround the heart surface.

Two views of the nominal geometries are shown for the three experiments are shown in green (torso) and black (heart) in Figure 8.

### 3.1. Additional details for the experimental datasets

We note that these three experiments were carried out at different times on three different animals, one using the cage and two using socks; for clarity we label these experiments in what follows as **cage**, **sock1**, and **sock2**. We describe further details about each of these experiments next.

**cage:** In this experiment the electrical activity of the heart was measured with a cage geometry containing 599 electrodes surrounding the heart and with 192 electrodes on the tank surface. The experimental procedure consisted of recordings during a series of ventricular pacings at four different sites followed by three series of ischemic episodes, as described above, all during sinoatrial pacing.

**sock1:** The sock used was outfitted with 247 electrodes and again there were 192 electrodes on the tank surface. Extra nodes, with no corresponding measurements, were added to the sock geometry to close the surface above the base of the ventricles, leading to a total of 337 nodes in the epicardial geometry mesh model. Ischemic interventions were interleaved with control periods; in this experiment there were 4 such ischemic episodes. All heartbeats during the experiment were paced at the sinoatrial node.

**sock2:** The sock, epicardial mesh model, and tank had the same dimensions as in **sock1**. The interventions consisted of an ischemic experiments with sinoatrial pacing followed by a series of ventricular pacings at five different locations. The specific sequence of interventions was: two initial series of sinoatrial paced control recordings, a series of ventricular pacings at various locations, and then a sequence of two ischemic interventions interleaved with control recordings, all under sinoatrial pacing. One important difference between this experiment and the others is that, after the first of the two initial series of control recordings, the heart was raised above the top of the tank and needle electrodes were inserted into the myocaridal wall (to be used for the ventricular pacing), and then the heart was lowered to the original nominal position. This difference plays a significant role in the results reported below.

**Preprocessing:** Before using the experimental data in both synthetic and experimental settings, we extracted the QRS complex from each heartbeat of both heart and torso surface recordings and applied a moving average filter of length 20 ms to both sets of signals to reduce noise. In the synthetic setting, only the heart surface recordings from one beat were used, while in the experimental setting all the data from both surfaces were used.

### 3.2. Computational procedure and validation details

We applied our geometry correction method to estimate the rotation and translation of the heart for each synthesized or recorded heartbeat. Given those estimated parameters we computed a corrected forward matrix for each heartbeat, synthesized the corresponding corrected BSP using the measured heart potentials as described in Equation (2), and computed two sets of inverse solutions using the synthesized / measured BSP and both the nominal and corrected forward matrices. We calculated inverse solutions using a zero'th order Tikhonov regularization solver (Equation 4).

(4) $$\begin{array}{r} \underset{x_{b}\left(t\right)}{\min}||y_{b}\left(t\right)-A_{b}x_{b}\left(t\right)|{|}_{2}^{2}+\lambda^{2}||x_{b}\left(t\right)|{|}_{2}^{2} \end{array}$$

where the norm was taken over both space and time within a single QRS. We used the L-curve method with 100 lambdas equally spaced between 10^−6^ to 1 on a logarithmic scale. We computed each point of the L-curve using all time instances within a beat to determine a single regularization parameter (λ) (Hansen, 2007) per beat.

Given these results, we calculated the relative error for beat *b* as the sum squared differences across all electrodes (*l*) and time instances (*t*) between measured BSP (*y*_*b*_(*l, t*)) and BSP synthesized using the corrected geometry (ŷ_*b*_(*l, t*)) divided by the sum-of-squares of the measured BSP (Equation 5).

(5) $$relErr_{b}=\frac{{\sum_{l}\sum_{t}\left(y_{b}l,t)-{\hat{y}}_{b}(l,t)\right)}^{2}}{\sum_{l}\sum_{t}y_{b}{\left(l,t\right)}^{2}}$$

To show the degree of improvement, we also computed the BSP relative error using the nominal geometry in the same fashion. Similarly, we computed the relative errors for the estimated cage/sock potentials for both corrected and nominal geometries. In the case of the synthetic experiment, where the true heart geometry was available, we also computed the root-mean-squared error (RMSD) between the true and corrected geometries as the square root of the average sum-of-squares of per-node errors (thus combining translation and rotation errors) across all nodes on the heart.

## 4. Results

**Figure 3:**
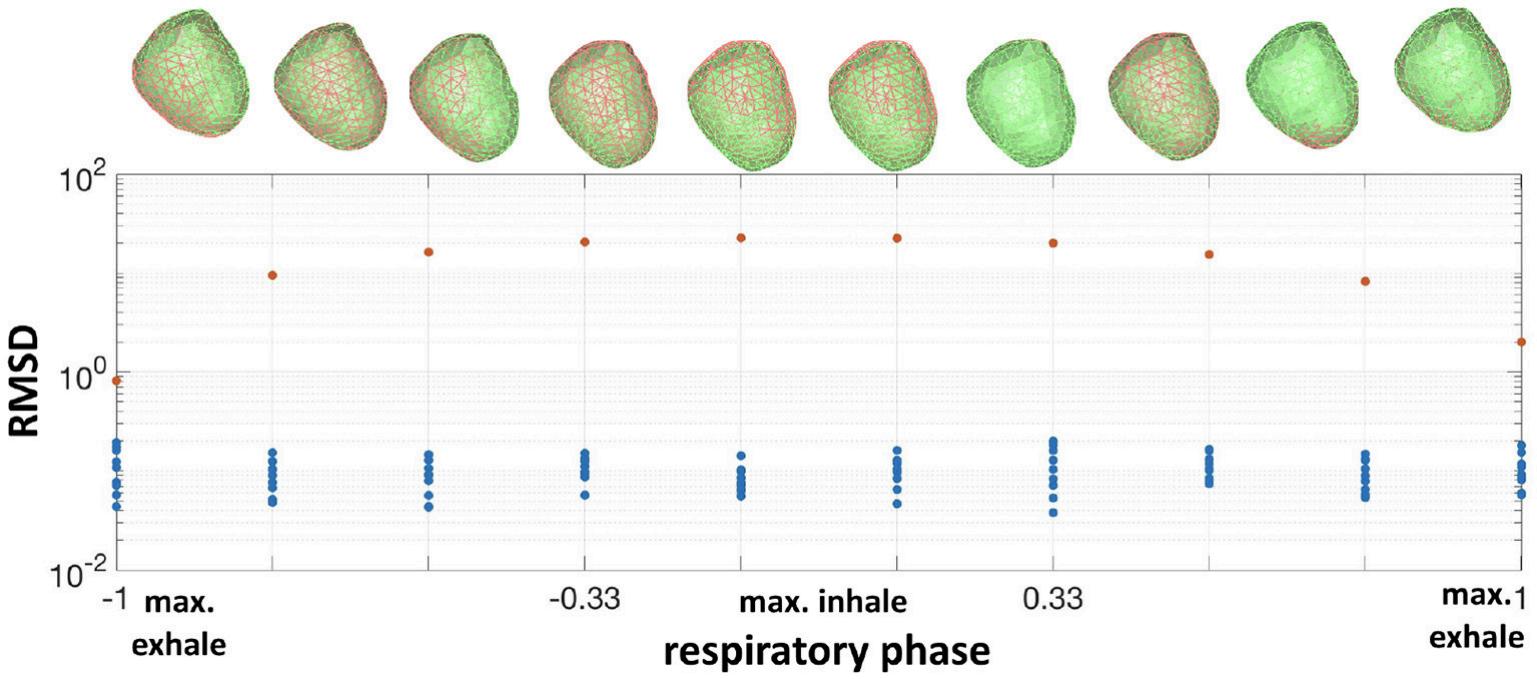
**Bottom**: RMSD of the nominal heart (red dots) vs. corrected (blue dots) for all respiratory phases. **Top**: corresponding true heart geometry (green) and corrected (red) for one repetition.The horizontal axis is normalized respiratory phase on a scale from −1 to 1.

In the **synthetic** experiments, as described above, we calculated the misplacement after correction at each time instant. The average RMSD after correction was 0.1±0.04mm, compared to an average error of 13.7±8mm before correction. To illustrate this result we plot the evolution of the RMSD and true and corrected heart geometries as a function of respiratory phase in Figure 3. As expected, the error of the nominal heart increased when approaching maximum inhale position, reaching 22.7 mm. This increase in error corresponds to the vertical displacement and slight rotation of the heart geometry. On the other hand, the RMSD for the corrected geometries was close to 0mm for most beats with a maximum RMSD of 0.19mm. This small RMSD can be observed in the almost indistinguishable true and corrected geometries shown in the figure.

**Figure 4:**
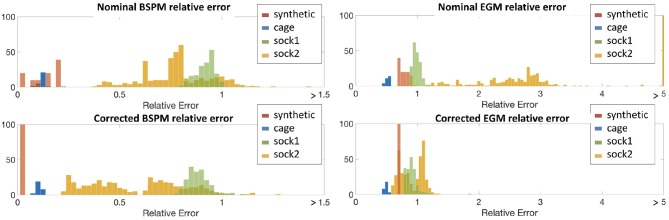
Histograms of relative errors for forward-computed BSPM (**Left**) and inverse-computed cardiac electrograms (EGM) (**Right**) potentials for all four experiments. Top row shows the relative error computed using the nominal geometry, bottom row using the corrected geometry. Data is shown from all four experiments in each panel: **synthetic** (red), **cage** (blue), **sock1** (green), and **sock2** (yellow).

We obviously cannot calculate actual misplacement for the three canine experiments, but we can study the differences between measured and synthesized signals both before and after correction. We plot these results in terms of relative error, as described above, in the form of histograms in Figure 4. To make comparisons easier, we used color to allow us to report all results for a single experiment on one plot. We report errors for both nominal geometries (top row of panels) and corrected geometries (bottom row) and for both errors in body surface potentials (left panels) and reconstructed EGMs (right). Color designates the specific experiment as shown in the legend. Each bar in the histogram shows the number of beats with relative error in the bin designated by the value on the horizontal axis at the position of the bar. So, for example, the red bars in the top left panel show that relative errors in the body surface potentials for the nominal geometry were distributed between 0 and 0.25, while after correction, in the bottom left panel, they were concentrated very close to zero, indicating the improvement after the correction.

From the top-left panel, we see that for the **synthetic** experiment the relative error between nominal and uncorrected forward-computed BSPM is rather evenly distributed between 0.0 and 0.17. For the **cage** experiment the relative error is in the same range and very stable across heartbeats—the average ± standard deviation BSPM relative error was 0.1±0.01. For **sock1** the relative error was again stable across all the recorded heartbeats but larger in magnitude (0.79±0.06). In contrast for **sock2** although the mean relative error was similar to **sock1**, the variability was considerably larger (0.8±0.25).

In the case of the inverse-computed EGM solutions, shown in the right column of the same row, the first notable observation about results from the nominal geometry is that the range of relative errors is, as might be expected due to ill-posedness, much higher than for the BSP. However within this range we note similar differences among results for the four experiments: a uniform distribution for **synthetic** data (0.76±0.06), lower mean error and small variability for the **cage** data (0.5±0.03), higher mean error but again small variability for the **sock1** data (1.3±0.39), and much higher variability for the **sock2** data (3.8±6.8). We also can observe that here the mean error for **sock2** was also higher than for **sock1**, in contrast to the results for the BSP's.

The bottom panels indicate a clear reduction in the relative error when geometry correction is applied. Numerically, the improvement in BSP relative error—measured as the difference between corrected and nominal relative error— was 0.07±0.04 for **synthetic**, 0.02±0.005 for **cage**, 0.1±0.02 for **sock1**, and 0.3±0.11 for **sock2**. The corresponding improvement in inverse-computed heart potential relative error was 0.067±0.06, 0.02±0.01, 0.4±0.4, and 3±6.4, respectively. Thus we see that the improvement is more pronounced for the inverse solutions than for the synthesized BSP, and greater in **sock2**. We also note that in general the improvement in inverse solutions, on average, accounted for much of the error we found using the nominal models.

**Figure 5:**
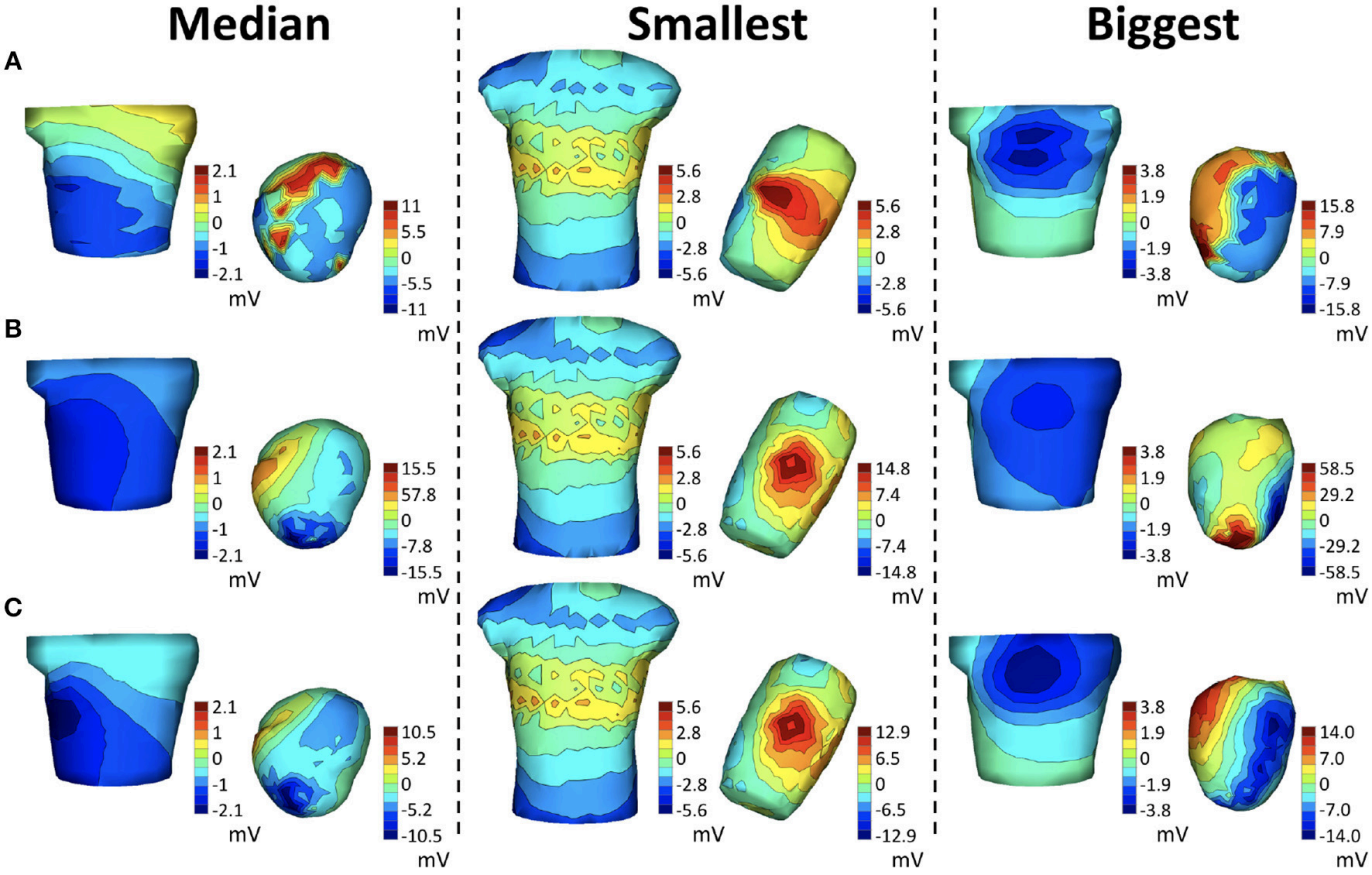
Potential maps of representative examples across all real experiments at QRS peak. The left column corresponds to the beat closest to the median BSP across all three canine experiments of the relative error improvement (0.18 in **sock2**), the middle column to the smallest improvement (0.006 in **cage**) and the right column to the biggest improvement (0.56 in **sock2**) across all experiments. Each row shows the BSP and EGM maps of the measured potentials **(A)**, maps synthesized with the nominal geometries **(B)** and maps synthesized with the corrected geometry **(C)**.

We show some illustrative potential maps taken as a snapshot at the QRS peak to give more insight into these summary results in Figure 5, which shows isopotential maps on the body and heart surfaces for representative beats. Maps of measured potentials are shown in the top row, maps of the nominal potentials in the middle, and maps of the corrected potentials in the bottom. The columns correspond to different example cases. The left column shows the heartbeat whose improvement in BSP relative error is closest to the median relative error of 0.18 across all three canine experiments, while the middle column shows the heartbeat with smallest relative error improvement, 0.006, and the right column shows the heartbeat with the biggest improvement, 0.56, again across all beats in all experiments. The median beat was sinoatrially paced and from **sock2**, the beat with biggest improvement is a ventricularly paced beat, also from **sock2**, and the smallest improvement beat comes from the **cage** experiment. Visually, the geometry correction provides a noticeable improvement for the “biggest” example beat, moderate improvement for the median example, and no obvious change for the “smallest” example.

**Figure 6:**
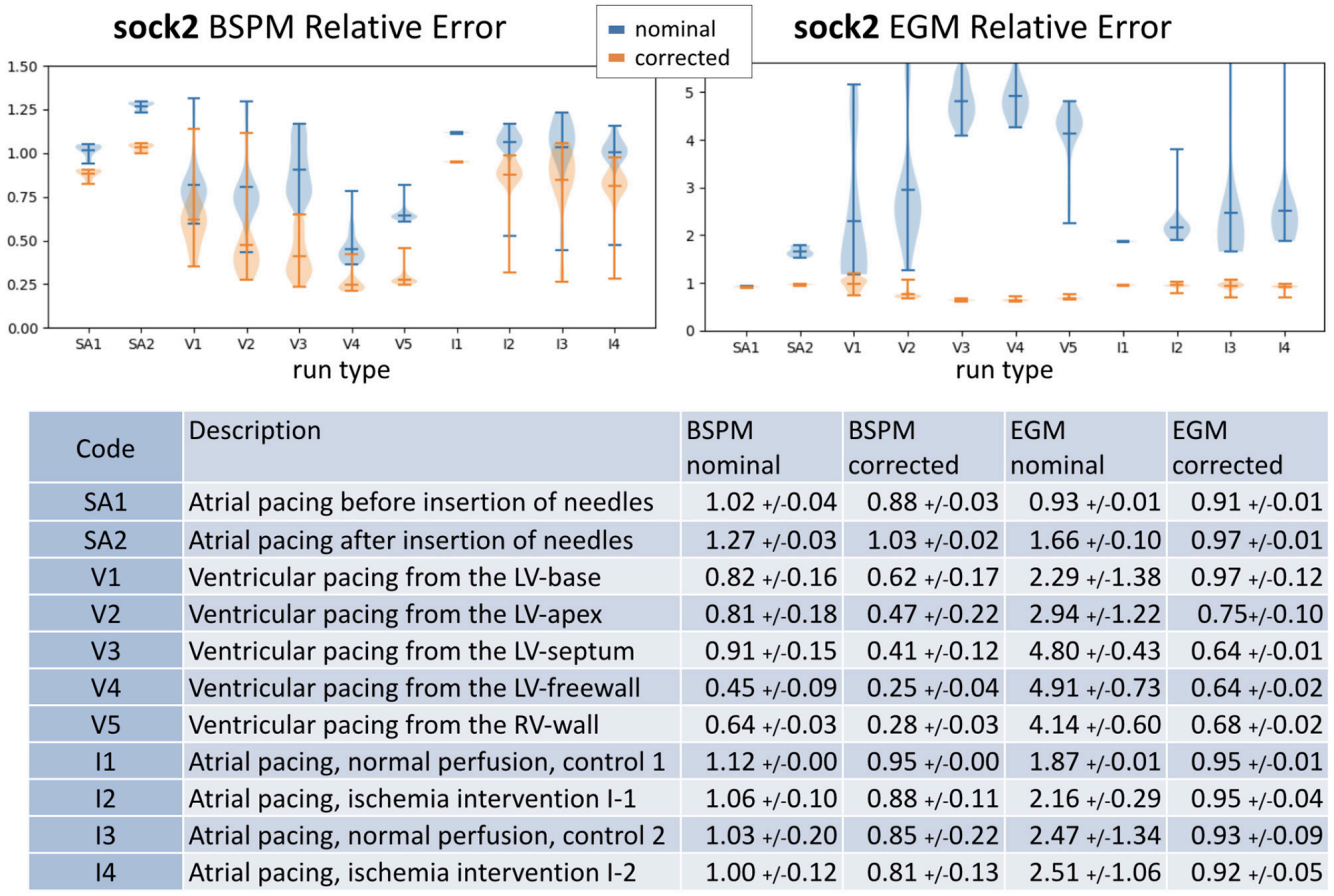
Summary of results for **sock2**. **Top**: whisker plots show relative error of BSPM **(Left)** and EGM **(Right)**. Blue whiskers correspond to relative error computed using the nominal geometry and orange using the corrected geometry. **Bottom**: the table contains a brief description of the type of intervention and summary statistics of the results.

Since the results for **sock2** were significantly more dramatic than those for **sock1** we examined the results from that experiment more carefully, as reported in Figure 6. We divided this experiment into into 11 consecutive stages that correspond to different control, pacing, and intervention epochs, briefly described in the table at the bottom of Figure 6. The errors summarized in the whisker plots at the top of the figure have considerable variability across all beats and interventions and decrease when using the corrected geometry. This decrease is more pronounced in the EGM inverse solutions, which appear to be very sensitive to the variations in the geometry. One noticeable result is that the first sequence of sinoatrial pacings—before the insertion of the needles—shows smaller relative error using the nominal geometries and correcting the geometry does not yield much improvement.

**Figure 7:**
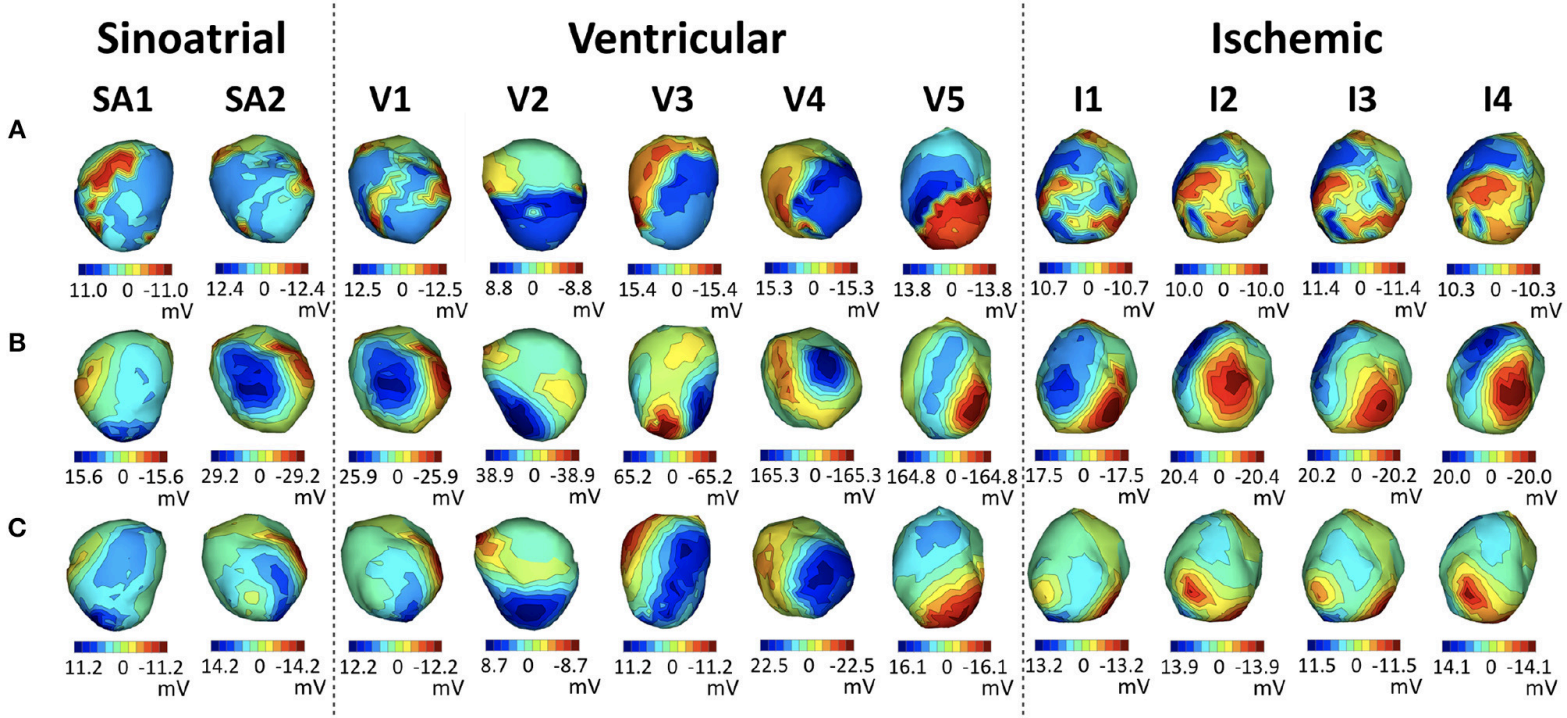
Representative examples of each stage of the **sock2** experiment. Each row shows EGM maps at QRS peak of the measured potentials **(A)**, synthesized with the nominal geometries **(B)** and synthesized with the corrected geometry **(C)**. Each column corresponds to a representative solution from each stage of the experiment, as described in the table in Figure 6.

In Figure 7, we show heart potential maps at peak QRS of a representative beat for each of the above stages of this experiment, as indicated by the headers using the codes from the table in Figure 6,^5^.

We note that in all stages after the insertion of the needles the inverse maps are more similar to the originals when the corrected geometry is used, and that the ventricularly paced beats show the most noticeable improvement.

**Figure 8:**
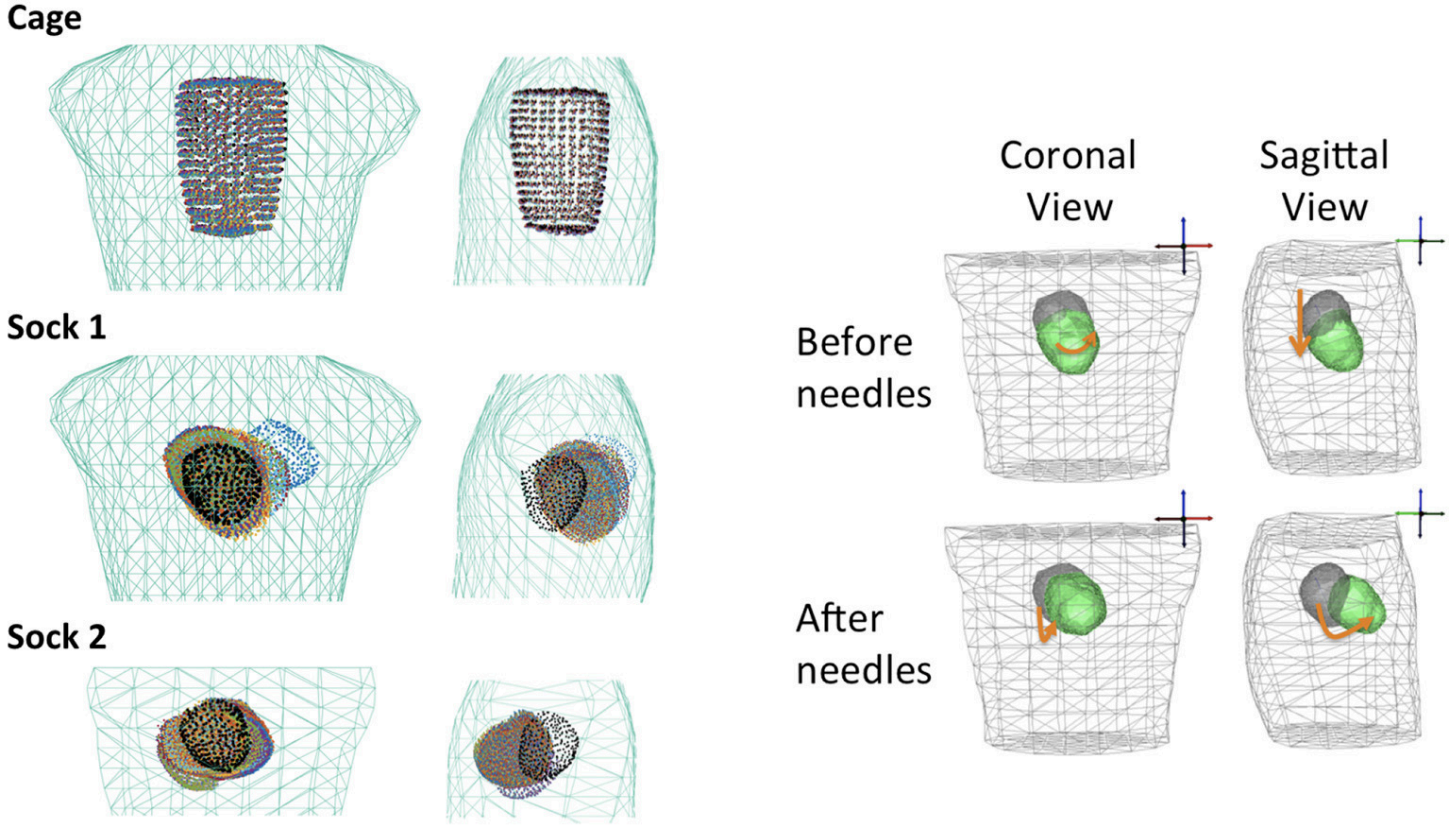
**Left:** Close-up of the nominal torso (green) and heart (black) geometries. The hearts corrected with the algorithm are overlaid in a different color per heartbeat. Top geometry is **cage**, middle is **sock1** and bottom is **sock2**. **Right:** Median position of the heart in **sock2** before the insertion of the needles (top) and after the insertion of the needles (bottom).

Looking at the reconstructed geometries themselves, in Figure 8 we illustrate the torso-tank geometries (in green), the nodes of the nominal heart geometry (in black) and the nodes of all corrected heart geometries (all other colors), from the three canine tank experiments. The behavior of the solutions varies depending on the experiment, although they cluster around a central location in each. As expected, the cage experiment does not show much change from the nominal geometry. The root mean square distance (RMSD) between the nodes of the nominal and corrected geometries is 4.5±0.8 mm. On the other hand, the sock experiments show considerable variability across heartbeats. Specifically, **sock1** has an RMSD of 22.6±4.8 mm and **sock2** 51.3±4.8 mm. The average translation and rotation of the θ angle—pitch of the heart—of the corrected hearts with respect to the nominal position are 20.3 mm and 19.1° for **sock1** and 37.2 mm and θ = 51.8° for **sock2**. Importantly, the heart in **sock2** has an estimated pitch rotation of ~50° after the insertion of the needles with respect to before the needles were inserted. To illustrate this change, Figure 8 shows the median estimated position and orientation of the heart before and after insertion of the needles in this experiment.

## 5. Discussion

The results presented above support the hypothesis that our method does improve the quality of the forward models. The method reliably corrected the heart geometry for the **synthetic** experiment, where ground truth was known, and provided considerable improvement in relative error of the inverse solutions. Moreover, although there is still unexplained error after applying our geometry correction to the real experiments, the estimated translations and rotations of the heart provide considerable improvement in both the synthesized BSP and all inverse solutions, in terms of both relative error and visual features of potential maps. In addition we note that inverse solutions improved notably even in some cases when the reduction in BSP error was small. The improvement was particularly strong for the **sock2** experiment, in which the broad spread of the error distribution of the inverse solutions with the nominal geometry was reduced to a much more concentrated one, similar to what was seen with **sock1**, after the correction.

We also observe that the positions of the heart estimated by this method are not randomly distributed throughout the torso, but rather show a physically meaningful structure: in particular, the largest correction factor is rotation near the anchor point above the atria. Moreover, the large rotation in **sock2** appears only after the insertion of the needles, suggesting that the needle cables could have been pulling the apex in an upwards direction. By contrast, the small change in the cage geometry experiment confirms the stability of the algorithm when the geometry is already accurate.These results suggest that the algorithm is detecting meaningful changes in position and orientation and not just overfitting to the noise.

On the other hand, there are some characteristics of the solutions in the sock experiments that are not consistent with the experimental setup. In particular there is a beat-to-beat variability around the central position that exceeds what might be expected, and the heart appears to be translated toward the edge of the bounding box of the optimization, well beyond what the experimental apparatus would permit. We believe that these errors are introduced by unmodeled sources of noise such as error in the shape of the heart and the lack of measurements around the atrial surface.^6^ Perhaps future developments, including estimating shape deformations as well as translation and rotation, as well as better characterization of the missing measurements (as was done in the cage experiment) could reduce these effects.

We also observe that although **sock2** showed considerable variability in the relative error of inverse solutions, the variability of the estimated position of the heart was relatively small (standard deviation of RMSD is 5.7 mm after the insertion of the needles). In fact, **sock1** showed much less variability in inverse solution relative error despite a similar standard deviation in RMSD (5.8 mm). The main difference between these two experiments was, however, in rotation correction, which was much larger for **sock2**, suggesting that rotation accuracy is a rather important factor in geometry model errors for ECGI.

In order to avoid constraining this approach to a specific forward solver, we used a black-box optimization method to solve Equation (3). For specific forward solvers and definitions of the geometry transformation, it should be possible to derive the corresponding gradients and Hessians for *A*(*p*_*b*_) such as in (Babaeizadeh and Brooks, 2007; Babaeizadeh et al., 2007) where we previously described how to compute Jacobians of both BEM and FEM models with respect to translation; Jacobians for rotation should also be possible to compute based on this work via the chain rule and appropriate rotation matrices.These analytical derivations should speed up the optimization and reduce the current computational demands of the method. However in the current work, since the computational demands were modest and easily within the scope of the Matlab solver we employed, and since as noted we preferred to be as general as possible in our presentation, we leave working out the details of such an approach to future work. However in cases with more densely sampled geometries, inclusion of more organs, or more complex source models, it might be necessary to explore alternative optimization approaches that are computationally less demanding. For example, in addition to analytically-based derivative computation, it may be useful to approximate the geometry with a smaller mesh or interpolate the manifold of forward matrices *A*(*p*_*b*_) with a continuous function that provides simpler analytic gradients and faster computation (Coll-Font, 2016). A second algorithmic consideration is that we are solving a non-linear optimization problem, which can have local minima. We have observed in our experiments to date that the nominal position of the heart is a good initial guess for global convergence using convex optimization solvers. However, this might not be applicable to all geometries and heartbeats and could be addressed, for example, by restarting the algorithm with different initial guesses or using global optimization techniques. An example of the latter that also addresses computational efficiency challenges is the class of Bayesian Optimization methods (Coll-Font et al., 2017), which carry out smart sampling of the unknown objective function based on a probabilistic representation that approximates it.

We want to point out that we used the zero-th order Tikhonov inverse method because it is so widely used for ECGI and its behavior is well-understood. However, this method tends to produce overly smooth inverse solutions with high relative error, even for ideal geometries, which may impact numerical results. Future work using other inverse methods might provide a better understanding of the interplay between regularization methods and geometry errors. Moreover, in settings where our simplified geometry assumptions—homogeneous torso and epicardial surface model—do not hold, we speculate that inverse solutions might show greater sensitivity to the accuracy of the position of the heart and thus benefit even more from the methods presented here.

A significant challenge for validation of our methodology is the lack of datasets with a reliable measurement of the real time-varying (e.g., from respiration) position of the heart. All existing ECGI datasets that we are aware of assume a static heart geometry and only provide a measure of its position at the beginning or the end of the experiment. Thus, existing datasets either have a highly accurate nominal geometry—which is not generally representative of clinical practice—or have geometry errors that pose a challenge to most ECGI methods. To better validate the method presented here, it would be helpful in the future to generate datasets with a continuous measure of the position of the heart using an external measurement modality such as with ultrasound.

Finally, an important follow-up to this work will be to incorporate estimation of the heart potentials along with the geometric changes, thus allowing extension of the scope of this method to clinical ECGI settings. Our initial work on this approach indicates that such an extension is possible and may provide useful results (Coll-Font, 2016; Coll-Font et al., 2017); a more extensive evaluation is currently underway.

## 6. Conclusions

In this work, we have introduced an approach to correct for the position and orientation of the heart inside the torso based purely on changes in electrocardiographic recordings along with an initial, nominal, geometry. We have shown that this approach can improve the forward models for both forward and inverse estimation of potentials and that it can provide useful insight for current experimental procedures used to validate ECGI methods. Moreover, the algorithm may be a first step toward solving the problem of joint estimation of the potential distribution on the heart and the heart's position and orientation within the torso.

We also add that our method may have implications beyond improving forward models in ECGI since the ability to non-invasively track the position of the heart might impact a number of other clinical problems, for example, improving catheter registration in ablation procedures.

## Ethics statement

This study was carried out in accordance with the animal treatment and ethical research recommendations of the IACUC at the University of Utah. The protocol was approved by the IACUC at the University of Utah.

## Author contributions

Both authors have made a substantial, direct and intellectual contribution to the work, and approved it for publication.

### Conflict of interest statement

The authors declare that the research was conducted in the absence of any commercial or financial relationships that could be construed as a potential conflict of interest.

## Footnotes

^1^Beyond the default configuration, we set the function and objective tolerance at 10^−6^ and extended the function evaluations to 10^10^

^2^In theory rotational symmetries of the heart geometry could produce non-uniqueness of the parameterization itself, but this is not a practical concern with a realistic heart shape, and in addition in the setting under study here we have access to the potentials on both surfaces, so that even rotational symmetries are removed modulo a highly improbable symmetry in the heart surface potentials as well as its geometry.

^3^The respiratory-like trajectory was interpolated from a time series of sagittal MRI scans of the torso at different phases of the respiratory cycle

^4^The SCIRun BEM solver is a standard BEM implementation for potential sources on an interior closed (epicardial) surface and measurements on an bounding closed (torso) surface, based on the classical “solid angle” integrations. Boundary conditions are the known epicardial potentials and an insulating boundary on the body surface; electrodes are treated as point electrodes with infinite impedance.

^5^In each case the illustrated beat was selected as the beat with BSP relative error closest to the median of each group

^6^The sensitivity of the solutions to the lack of measured data on the atria can be taken as another indication that solutions are indeed sensitive to the measurements, that is, that the problem is reasonably well-posed.
